# Towards Differentiated Management: The Role of Organizational Type and Work Position in Shaping Employee Engagement Among Slovak Healthcare Professionals

**DOI:** 10.3390/healthcare14010007

**Published:** 2025-12-19

**Authors:** Veronika Juran, Stela Kolesárová, Viktória Ali Taha

**Affiliations:** 1 Faculty of Management and Business, University of Prešov, 080 01 Prešov, Slovakia; 2 Rectorate UPJŠ, Pavol Jozef Šafárik University in Košice, 040 01 Košice, Slovakia; viktoria.ali.taha@upjs.sk

**Keywords:** healthcare workers, engagement antecedents, well-being, differentiated management, HRM

## Abstract

**Background/Objectives**: Employee engagement is fundamental for the quality and sustainability of the Slovak healthcare sector. While the concept is critical, its operational challenges lie in the differentiated perception of its drivers across the highly heterogeneous workforce. This study aimed to empirically identify and structure the key antecedent factors of engagement and examine their perception based on structural and sociodemographic characteristics among healthcare workers in Slovakia. **Methods**: This research employed a quantitative, cross-sectional design, utilizing a self-administered questionnaire distributed widely among healthcare providers throughout Slovakia. To achieve the study’s objectives, several advanced mathematical and statistical methods were applied: the Kaiser-Meyer-Olkin (KMO) Measure and Bartlett’s Test for sample adequacy, Principal Component Analysis (PCA) for empirical factor structuring and Analysis of Variance (ANOVA). **Results**: Three common antecedent factors for healthcare workers’ engagement and well-being were identified: Factor 1—Organizational Commitment and Identity; Factor 2—Meaningful Involvement and Job Satisfaction; and Factor 3—Organizational Citizenship and Retention Intent. Factor 1 was evaluated positively in public (state-owned) and mixed organizations but negatively in private healthcare providers, confirming a statistically significant difference. Factor 2 also exhibited significant differences based on work position: it was negatively rated by management, physicians, and nurses, but positively by other staff categories. **Conclusions**: The contribution of this study lies in the empirical confirmation that a universal managerial approach to increasing employee engagement in Slovak healthcare is ineffective. A differentiated managerial approach based on organizational type and work position directly supports the transition from blanket, expensive, and ineffective HR policies to strategic and targeted engagement management, which is essential for the long-term sustainability and improvement of care quality in Slovak healthcare.

## 1. Introduction

The healthcare system is a serious social, economic and political issue worldwide. Many countries are attempting to reform their healthcare systems due to dissatisfaction with the level or manner of care provided for various reasons [1]. The aging population, together with an increase in chronic and age-related diseases, is leading to an increase in demand for healthcare. Healthcare systems are facing rising costs due to constant technological development and growing competence requirements. Furthermore, inequalities exist primarily in access to healthcare [2]. Given these systemic challenges which globally affect the sustainability and quality of services, it is essential that management in this sector focuses on efficiency and process optimization. An effective healthcare system contributes to the well-being of the population and to economic growth. Despite the size and importance of this sector in structural policies, there is still space for improvement. Advanced economies tend to invest more in healthcare and often at a faster rate than their economy grows, which is also the case in Slovakia. In 2024, public spending on health insurance reached 6.5% of GDP. According to the latest available comparable data, which does not consider pandemic spending, Slovakia spends more on healthcare than the average of other V4 countries, namely 5.9%, not less than the EU14 (7.8%). Compared to 2015, healthcare spending increased by almost 90% by 2024, while GDP grew by 64% over the same period [3]. One of the greatest challenges facing Slovakian healthcare is workforce. Slovakia faces a long-term shortage of healthcare personnel, especially nurses, while a high-quality healthcare workforce is an essential prerequisite for the quality and availability of provided healthcare, as well as patient satisfaction. In inpatient healthcare (hospital care), there is a deficit of almost 6000 healthcare workers, representing approximately 10% of the total required workforce in 2023 [4].

Healthcare workers engagement and retention are considered key factors for high quality, patient safety and ensuring long-term sustainability in the context of a demanding and constantly changing healthcare sector [5]. Higher engagement of healthcare workers is in the literature associated with better patient care outcomes, lower stress levels, higher job satisfaction and reduced risk of burnout [6,7,8]. Engaged and satisfied employees represent active partners in the healthcare process, as they provide better patient care, manage crisis situations more effectively and make fewer errors, ultimately increasing patient safety [9,10,11,12]. The healthcare sector is instantly under pressure, marked by staff shortages, high workloads, stress and concerns about burnout [13,14]. These global challenges are intertwined with local specificities specific to each country and its healthcare system—including, for example, the availability of resources, management practices and legislation in Slovakia, which influence employee satisfaction and loyalty [5,15]. As a result, greater emphasis is placed on understanding the factors that support or hinder employee engagement in this year-round context [16]. In this context, engagement refers to the emotional, behavioural and mental employee involvement in their work, driven by intrinsic motivations for healthcare and a strong sense of organizational commitment [9,17,18,19]. Healthcare workers well-being is no longer simply the absence of an employee due to a long-standing illness, but precisely defined as a holistic state of physical, mental and social health that allows creativity to flourish and contribute effectively to the values of organizations [20]. As a driver, work engagement is a strong indicator of psychological well-being, acting as a resilience that protects employees from chronic stress and burnout [21,22,23,24,25]. Overall job satisfaction is a key indicator of job well-being, confirming the same relationship between higher engagement, greater satisfaction and improved employee health [26,27]. The antecedents of engagement fall into two main components: organizational and individual. To retain staff and increase engagement, healthcare facilities implement key organizational practices. Organizational antecedents of engagement include [28,29]: (1) Effective leadership and support: This includes clear communication, support from management and the use of leadership styles [30,31]; (2) Participatory and collaborative practices: Promoting participatory decision-making, more effective teamwork and a sense of belonging and responsibility [32]; (3) Development and Learning: Providing access to development and training (including new technologies), recognition, rewards for performance, and opportunities for professional growth [33,34]; (4) Supportive Environment: Implementing flexible work arrangements and mental health support programs in response to high workload and stress [31]. Individual prerequisites include the necessary competencies, intrinsic motivation, resilience, and willingness to participate in team activities [35,36]. Studies support that high satisfaction contributes to organizational stability and loyalty, directly reduces turnover, and positively affects team well-being [7,37,38].

Therefore, identifying specific factors that support engagement is a key strategy for the long-term sustainability of the entire system. In addition to primary factors related to workload and work organization, it is crucial to consider individual and structural (sociodemographic) variables when assessing the engagement and well-being of healthcare workers. Studies repeatedly confirm that these factors significantly modulate resilience to stress and the perception of the work environment. The influence of sociodemographic factors on the occurrence of professional burnout symptoms among nurses was confirmed in Poland. Among the most frequently cited factors were age, gender, education, marital status, and number of years worked [39]. Similarly, sociodemographic factors significantly influenced the prevalence of depressive disorders within another study group. A strong relationship was confirmed between gender, age, and the depression index [40]. Study on employee motivation in healthcare and other sectors identified significant differences in motivational factors among Slovak employees depending on their gender and level of education. The most important motivational factors identified were basic salary, job security and a good work team [41]. A study focused on the antecedents (preceding factors) of Health-Oriented Leadership (HOL) from the leaders’ perspective identified various organizational, job, and individual factors influencing its implementation. Crucially, the findings demonstrated that barriers specific to the healthcare sector, alongside organizational size, significantly impact the overall perception and effective practice of HOL [42]. Another study focused on work engagement and its dimensions among hospital employees yielded that the work engagement and its individual dimensions were significantly higher in doctors than in nurses [43]. This observation may be partially explained by the fact that the literature repeatedly identifies high levels of workload among nurses across different forms of healthcare provider organizational type (whether public or private). Factors that most strongly influence subjective perceptions of workload include time pressure, high levels of responsibility, and perceptions of long-term work sustainability [44,45]. Although most studies have traditionally focused on nurses in institutional care, in inpatient wards in public hospitals, there is growing evidence suggesting comparable burden among nurses working in the private sector. Some studies have even reported higher rates of perceived burden and psychosomatic distress among outpatient staff in private clinics [46]. The inclusion of these structural and sociodemographic variables in the analysis is therefore essential to accurately identify and differentiate risk working groups and to propose targeted managerial and preventative interventions. In the context of personnel shortages, reducing turnover and enhancing productivity are key. Healthcare managers should help their workers realize the meaning and value of their work and encourage active commitment to their work through incentive mechanisms [47]. Therefore, the application of differentiated HRM could represent one such mechanism for fostering higher employee engagement, especially in under-resourced sectors like healthcare. However, empirical evidence regarding the applicability and effectiveness of differentiated HRM within the specific context of the Slovak healthcare system remains limited, highlighting a crucial gap in current research. Comprehensive research that systematically identifies and empirically groups the factors that most influence healthcare workers’ engagement and well-being in the local environment is missing. Although these preconditions are generally recognized as essential for ensuring quality, safety, and long-term sustainability within the healthcare sector, current scientific literature reveals a significant deficit within the Slovak context. Furthermore, there is also a lack of rigorous analyses examining if and how the perception of these critical antecedents differs across various structural (organization type, size and work position) and sociodemographic categories (age, gender, education) in Slovakia. Current literature mostly adheres to a universalist approach, thereby overlooking the fundamental structural and demographic variability within the Slovak healthcare system Specifically, we lack knowledge on how to translate general managerial approaches into differentiated management practices. Closing this gap is essential if we want to move from descriptive findings to prescriptive differentiated management approaches.

## 2. Materials and Methods

The aim of the research is two-fold: first, to identify and empirically structure the key antecedents of employee engagement and well-being into underlying factors within the Slovak healthcare sector; and second, to examine whether the perception of these factors significantly differs based on selected structural and socio-demographic characteristics (gender, age, education, organization size and type, and work position). To achieve the two-fold aim of the study the two main research questions were stated:

RQ1: What are the key dimensions of employee engagement and well-being antecedents relevant to healthcare workers in Slovak context?

RQ2: Which structural and socio-demographic variables significantly influence the perception of the identified key factor dimensions of engagement and well-being antecedents among Slovak healthcare workers?

In order to achieve the study’s secondary objective of comparing the perception of these factors based on structural and sociodemographic characteristics, the following research hypotheses were formulated:

**H1.**  *The perceptions of the identified antecedent factors vary statistically significantly across gender*.

**H2.**  *The perceptions of the identified antecedent factors differ statistically significantly across age groups*.

**H3.**  *The perceptions of the identified antecedent factors vary statistically significantly across educational levels*.

**H4.**  *The perceptions of the identified antecedent factors differ statistically significantly based on organizational size*.

**H5.**  *The perceptions of the identified antecedent factors vary statistically significantly across organization type*.

**H6.**  *The perceptions of the identified antecedent factors differ statistically significantly based on work position*.

The paper is divided into three main stages: (1) analysis of the research sample; (2) identification of the factors that affect employee engagement antecedents and well-being; and (3) examination of the structural and socio-demographic characteristics that influence the perception of these factors in the healthcare sector.

In order to ensure the content and convergent validity of the research tool (questionnaire), items (variables) related to engagement were taken from existing, validated, and reliable scales or tools used in practice to measure employee engagement [48]. To construct a coherent and theoretically grounded measure of employee engagement and well-being, we began with a pool of items drawn from four frequently cited and psychometrically established engagement instruments: (1) Employee engagement scale [49]; (2) JRA Employee Engagement Scale [50]; (3) Job Engagement Scale [51]; (4) Psychological Engagement Measure [52]. These instruments were chosen because, taken together, they represent the dominant conceptualizations of engagement—cognitive, emotional, behavioral, and psychological safety/availability—presented in the literature. Given the breadth of the initial pool, we proceeded with a structured item-reduction process. Our goal was to balance conceptual coverage with respondent feasibility and to minimize redundancy across overlapping constructs. The selection criteria were threefold: (1) theoretical alignment with our definition of engagement as a multidimensional, motivational state, (2) empirical robustness, privileging items with strong loadings, internal consistency, and demonstrated reliability in previous validation studies, (3) clarity and relevance for our applied healthcare context. Final version of a questionnaire has the following structure: the introduction provided background information was followed by identification items to collect basic demographic data about the respondents and the organizations they work for. The core consisted of 18 factual items (in the form of statements) divided into four groups: (1) items focused on antecedents (prerequisites) of engagement, (2) items related to cognitive engagement of employees, (3) items related to emotional engagement of employees, (4) items related to the behavioural engagement of employees. Finally, there was a question about barriers to engagement, which was a polytomous calculation question in which respondents were asked to identify the five most significant barriers to engagement.

Healthcare workers were contacted via email using a random selection of addresses from the national online healthcare portal zzz.sk, which maintains a publicly available list of 14,536 healthcare facilities across Slovakia. From this database, 1134 recipients were randomly selected and invited to participate in the study. The invitation email included essential information about the research purpose, ethical assurances, and a direct hyperlink to the questionnaire. This method ensured that every potential participant within the sampling frame had an equal probability of being contacted, preserving the logic of random selection at the initial stage. However, it is generally known that healthcare professionals are among the low responsive groups in voluntary survey research. Their demanding clinical responsibilities, long shifts, and unpredictable workloads substantially limit the time available for non-mandatory tasks such as filling out questionnaires. Additional factors—such as digital fatigue, information overload, and institutional pressures—further discourage participation. Despite these limitations, the obtained sample displays heterogeneity across age, tenure, and healthcare roles, which supports the analytical aim of examining relationships between variables. Moreover, the main purpose of the study was theory-driven analysis of the psychological mechanisms of engagement rather than drawing national inferences about the absolute levels of engagement. It is also important to note that obtaining truly nationwide, population-level data in the healthcare sector is extremely challenging for individual researchers, as even national institutions routinely face difficulties securing comprehensive participation from healthcare workers.

For the purpose of identifying and verifying statistically significant relationships, several mathematical and statistical methods were employed, including Kaiser-Meyer-Olkin (KMO) Measure of Sampling Adequacy, Bartlett’s Test of Sphericity, Principal Component Analysis, and Analysis of Variance (ANOVA).

## 3. Results

The research sample comprised 186 healthcare professionals consisting of 132 female and 54 male participants. Regarding professional roles, the sample comprised diverse positions within healthcare. The most represented were nurses (30.8%), followed by doctors (24.6%). Other roles included middle and lower management representatives (e.g., department heads, section chiefs) (10.5%), top management representatives (e.g., directors, deputies) (8.4%), administrative staff (8.4%), laboratory assistants/technicians (8.4%), and pharmacists (8.9%). This distribution reflects a range of responsibilities and hierarchical levels within the participating organizations.

The research sample included participants working in the healthcare organizations of different sizes. Most of respondents were employed in large organizations with 251 or more employees (64.5%), followed by medium-sized organizations with 51 to 250 employees (18.8%), and small organizations with 0 to 50 employees (16.7%). Participants also represented different types of organizations. Over half of the respondents worked in public state-owned organizations (55.8%), while 30.4% were employed in private owned organizations. Organizations with mixed ownership accounted for 13.8% of the sample.

Based on the value of the Keiser-Mayer-Olkin statistic (0.915) and according to the definition according to Kaiser, it can be said that the correlation rate is good and that the choice of PCA for the analysis of engagement assumptions based on the research tool used is justified. The achieved significance value of Bartlett’s test of sphericity (*p* = 0.000) is smaller than chosen significance level α = 5% and therefore we can reject the null hypothesis that the realization of the selection correlation matrix with 18 considered variables is a unit matrix (Bartlett’s Test of Sphericity: Approx. Chi-Square = 1786.365; df = 120; *p* = 0.000). It can be concluded that the analysis we have chosen is suitable for the analysis of data dealing with the engagement assumptions of the entire research set. To assess the reliability of the research tool used, the Cronbach’s alpha coefficient was applied, and its obtained value was 0.869260. Since the value of Cronbach’s alpha exceeds 0.7, we can conclude that the research tool is reliable and that the data obtained using it can be further worked with. The table of eigenvalues of the source matrix (Table 1) shows that the first main component, or the first factor, concentrates 54.74149% of the variability of the original data, the second principal component 7.0624%, the third component 5.662%, and the last, eighteenth component 0.91841%. The first three main components, whose eigenvalue is greater than 1. concentrate 67.4659% of the variability of the original data of the entire research set. These results will subsequently be used for the description and identification of factors within the application of factor analysis.

Table 2 indicates that the initial factor derived from the principal components analysis exhibits a substantial correlation with the antecedents of employee engagement PA_8. PA_9. PA_10. and PA_12. The values of the factor loadings reached the value of 73.7008% for PA_8 to 80.2443% for PA_9. The analysis of factor 1 revealed that the first common factor explains 54.31% of the variability of the engagement antecedent PA_8. 64.39% of the variability of the engagement antecedent PA_9. 58.68% of the variability of the engagement antecedent PA_10. and 54.93% of the variability of the engagement antecedents PA_12. The second common factor correlates with these antecedents of employee engagement: PA_4. PA_5. PA_6. and PA_7. The values of the factor loadings range from 74.7626% at PA_6 to 76.8509% at PA_7. From the analysis of factor 2. we further see that 56.45% of the variability of the engagement antecedent PA_4. 57.28% of the variability of the engagement antecedent PA_5. 55.89% of the variability of the engagement antecedent PA_6. and 59.06% of the variability of the engagement antecedent PA_7 is explained by the second common factor. The third common factor correlates with the of the engagement antecedents PA_15 and PA_18. The values of the factor loadings represent the values of 70.9121% for PA_15 and 78.0606% for PA_18. In this way, we simultaneously verified the guarantee of practical significance of the factors. From the analysis of factor 3, we further see that 50.28% of the variability of the engagement antecedent PA_15 and 60.93% of the variability of the engagement antecedent PA_18 is explained by the second common factor. The total number of variables (antecedents of engagement) was reduced to three common factors. Based on our results, it can be observed that the key dimensions of employee engagement and well-being antecedents relevant to healthcare workers in the Slovak Republic are the following factors:

- Factor 1: Organizational Commitment and Identity.
- Factor 2: Meaningful involvement and Job satisfaction (the factor corresponds to Kahn’s psychological meaningfulness);
- Factor 3: Organizational Citizenship and Retention Intent.

**Figure 1:**
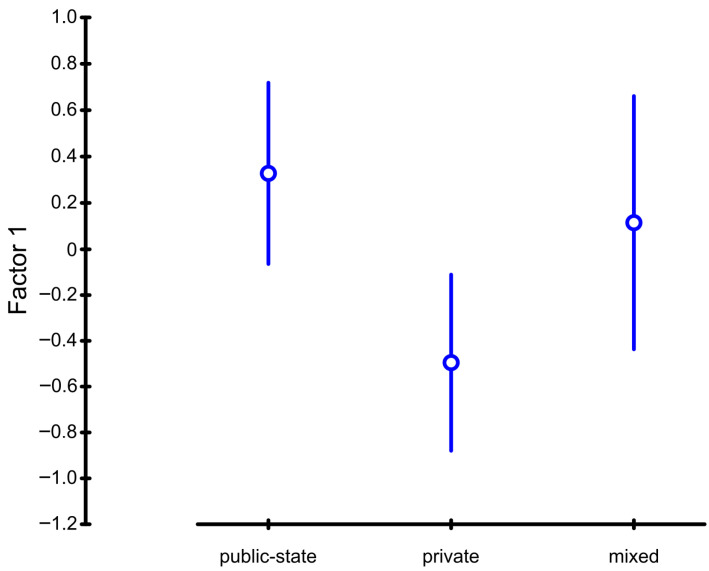
Factor Score Plot for Factor 1 and Type of the Organization. Source: own elaboration.

From the average value of the factor score for individual categories, Factor 1 is positively evaluated by respondents in public state-owned healthcare providers and in organizations with mixed ownership. In private-owned healthcare providers (ambulances and clinics), this factor was evaluated negatively. It is therefore possible to state that at the significance level of 5%, the mean values of the factor scores are statistically different between these individual types of organizations. These results allowed us to accept the fifth hypothesis (Table 3 and Figure 1).

**Figure 2:**
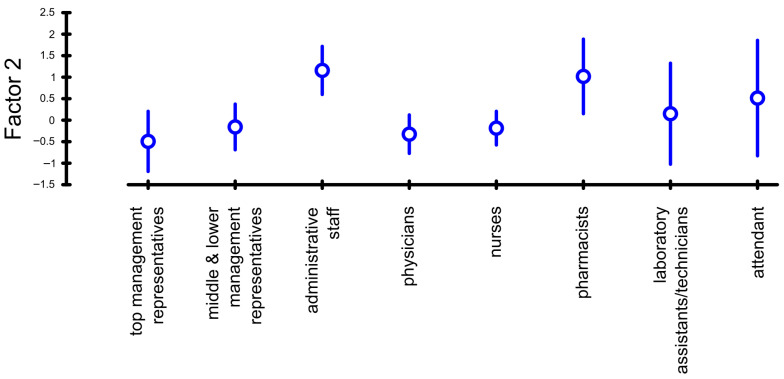
Factor Score Plot for Factor 2 and Work position. Source: own elaboration.

It is possible to conclude that at the selected level of significance of 5%, the mean values of the factor scores are statistically different between the individual work positions held, and thus the work position significantly affects the evaluation of Factor 2. Based on these results, we were able to accept the sixth hypothesis. From the average value of the factor score for individual categories, it is showed that Factor 2 is negatively evaluated by representatives of top and middle management, physicians, and nurses. Other employees’ categories evaluated this factor positively (Table 4 and Figure 2).

Regarding Factor 3, which represents organizational citizenship and retention intent, no statistically significant differences were found between the individual groups. Thus, the studied socio-demographic variables do not have a statistically significant effect on the change in the value of the factor score for Factor 3 (Table 5).

The results of our analysis indicate that two of the six formulated research hypotheses were supported (H5 and H6). Within these hypotheses, we assumed a difference in the perception of key antecedents depending on the type of organization and the employees’ work position.

## 4. Discussion

This study’s findings advanced the knowledge base concerning engagement and well-being among healthcare professionals in Slovakia. Specifically, the empirically derived three-factor structure of the antecedents, coupled with the uncovered striking sociodemographic differences, yield crucial data that directly facilitates a critical comparison with results obtained across various international healthcare delivery systems:

### 4.1. Factor 1: Organizational Commitment and Identity

Factor analysis revealed significant differences in the perception of organisational commitment and identity across the organisational landscape. The variations measured within the study groups were found to be highly statistically significant (*p* < 0.05).

Key findings revealed significant differences in the perception of organisational commitment and identity across the healthcare organisational landscape. This result, therefore, supported the fifth stated hypothesis (H5). Employees from public and mixed healthcare providers viewed affirmation and identity favourably, while respondents from private healthcare providers (i.e., clinics and outpatient clinics) offered a decidedly negative assessment. The literature support for the finding that healthcare employees in the public sector generally show higher organizational commitment than those in private healthcare facilities, primarily due to greater job security and employment stability, already exists [53,54]. However, local context and specific organizational factors can influence these outcomes.

The finding that public and mixed entities in Slovakia rate their commitment highly while private entities pull the average down is a strong indicator of potential differences in underlying organisational culture and day-to-day management. Compared to publicly owned healthcare providers with a long history, a deeply rooted and transmitted organizational identity and mission is not possessed by private entities, which complicates the creation of organizational commitment with healthcare workers. The process of building this organizational commitment calls for the application of differentiated managerial approaches.

Due to the specific presence of strong profit orientation in the private sector, constant pressure is exerted on employees, which reduces staff well-being. Ultimately, the consequence is a decline in the quality of patient care. It is therefore recommended that employers ensure adequate staffing levels, provide structured breaks for recuperation, and implement psychological support programs to prevent burnout. To boost organizational commitment, the private sector must complement its competitive, fair salaries with personalized rewards for extra effort and a strong focus on employee well-being benefits (such as sports contributions or supplementary insurance), which are crucial for enhancing stability perception. Within smaller private healthcare providers, focus should be placed on building an organizational culture based on cooperation, trust, teamwork, and warm interpersonal relationships. Its establishment can be supported by regular (including informal) joint activities and shared rituals. Persistently unsatisfactory ratings in private healthcare settings should serve as a major warning sign, requiring urgent remediation and targeted corrective action. Ongoing negative feedback from the private sector requires in-depth investigation and strategic peacebuilding to improve internal communication. Private healthcare facilities with centralized management often foster a feeling among employees that their opinions are unheard, leading to low participation and engagement. To remove these barriers, it is crucial to implement also participatory management practices that incorporate employees into decision-making processes and ensure clearer communication about their contribution to the organization’s overall results and strategic goals. As care is context- and situation-specific and tailored to circumstances, it is important to address employees’ immediate needs, which may vary depending on the setting, e.g., the workplace, education, counselling or professional care. Therefore, it is important to empirically research existing theoretical frameworks further, as these could be expanded and deepened, especially with regard to the context of care [55]. According to Lee [56], who investigated the influence of organisational culture and capabilities on employee commitment to ethical behaviour in the healthcare sector, a well-functioning organisational culture was found to motivate employees to perform better through collaboration and ethical work behaviour. Other studies emphasise the dual impact of organisational identification and reinforce the need for organisations to cultivate employee engagement while instilling ethical leadership and clearly defined behavioural standards [57,58]. Care management studies involve analysing and understanding employee well-being, the importance of which in organisations has been recognised for many years. Recently, however, the concept of care has received increasing attention in the fields of business, social policy and academic research, as evidenced by a growing number of publications [59,60,61,62].

Caring, sharing and acting for the benefit of others are considered deep moral values in most societies [63,64]. In practice, however, several studies have shown that many organisations express concern for the well-being of their employees without fulfilling it [65,66,67]. Therefore, it is essential to build organisations that actively promote mutual support and a sense of community, and that are not afraid to ‘care’. Such organisations should be inspired by a deep understanding of human vulnerability and have the principle of care rooted directly in their organisational DNA [68,69]. The study of employee care also significantly contributes to the development of humanistic management, a field gaining increasing importance in contemporary organisations. All these factors encourage a deeper analysis of care management and its implications for research on the institutionalisation of employee care.

### 4.2. Factor 2: Meaningful Involvement and Job Satisfaction

The finding that Meaningful Involvement and Job Satisfaction emerged as a separate factor strongly underpins theories of motivation and personal contribution to work. Specifically, this factor directly links to the critical concept of psychological meaningfulness introduced by Kahn [70]. This study defined psychological meaningfulness as the feeling that work is valuable, useful, and meaningful, which is a key prerequisite for employees to be fully engaged in their work. Our empirical clustering related to involvement and satisfaction confirms that Slovak healthcare workers prioritize the intrinsic value of their work. From a well-being perspective, this is a key criterion: when healthcare professionals perceive their roles as deeply meaningful, it acts as a vital psychological resource that not only drives engagement (a motivational process) but also contributes fundamentally to eudaimonic well-being—a sense of meaningfulness and self-actualization.

According to Benishek et al. [71], meaningfulness is one of the three basic psychological prerequisites (along with psychological safety and availability) that must be met for an employee to fully invest their personal involvement in a work task, i.e., to express themselves physically, cognitively and emotionally at work. Statistically significant differences in the perception of the meaningful involvement and job satisfaction factor were identified across different work positions; thus, the sixth hypothesis (H6) was supported. Consequently, the necessity for differentiated human resource management approaches in addressing these issues is underscored. A surprising lack of perceived meaningfulness was found among senior and middle-level managers, as well as physicians and nurses, as reflected in the average score. Conversely, employees in all other operational categories expressed positive sentiment. These results highlight a clear dichotomy in the overall work atmosphere, indicating that an individual’s position in the hierarchy significantly impacts their perception of meaningfulness at work. This makes perceived meaningfulness a critical area for organisational focus. The lower meaningfulness observed among management and clinical staff may be due to high levels of responsibility, excessive job stress or a lack of recognition for their work, which can negatively affect their motivation and loyalty. High stress levels in medical practice can significantly impact the well-being of physicians, the quality of care provided and the overall functioning of the healthcare system. For instance, studies from China indicate that, in environments with high service demand, physicians frequently encounter excessive workloads and extended working hours, resulting in work-family conflict (WFC) [72,73]. Examining the internal mechanisms of this relationship is important, as factors such as job satisfaction and work engagement play a particular role in mediating the impact of work-family conflict (WFC) on physicians’ well-being [74,75]. To enhance meaning and job satisfaction, Slovak healthcare managers should focus on addressing the specific workload inequity among nurses through transparent shift assignment and simultaneously investing in their continuous professional development. The meaningfulness of work can be perceived by every employee primarily based on its results; therefore, it is important that the direct impact of their work on patients’ lives is perceived by physicians. The sharing of successful clinical case studies and testimonials regarding patients who were helped by physicians should be presented at internal meetings and in circulars to remind hospital management of the direct impact of their work. Increasing the perception of meaningfulness and job satisfaction among top and middle management is recommended to be achieved through regular time spent directly on the wards, in outpatient clinics, or in the field. Managers will thus be enabled to regain a sense of meaningfulness in their work through a visible connection to reality.

Furthermore, the job satisfaction, engagement, well-being and performance of healthcare workers are influenced by multiple factors. Salary is often identified as a major source of dissatisfaction and is among the most important factors; research confirms that a higher salary significantly improves performance in the healthcare sector [76,77]. Previous studies on job satisfaction have shown that perceptions of justice and autonomy, as well as overall employee well-being, can mediate the relationship between job satisfaction and job performance. At the same time, well-being is a key factor that significantly influences satisfaction, engagement, and performance [78,79,80].

### 4.3. Factor 3—Organizational Citizenship and Retention Intent

Analysis of the results revealed no statistically significant differences in Factor 3 scores across the examined sociodemographic variables. This suggests that perceptions of these specific aspects are consistent across demographic groups within the sample of Slovak healthcare workers, indicating stable perceptions of the work environment. These findings are consistent with the theoretical framework of organisational citizenship behaviour (OCB), which refers to voluntary employee behaviour that goes beyond their formal job responsibilities. OCB is considered highly important in organizational effectiveness and well-being research. OCB encompasses activities such as assisting colleagues, actively engaging in team activities, surpassing job expectations, and fostering a positive work environment, as defined by Gan and De Geus [81,82]. The literature mainly attributes the importance of OCB to its contribution to better collaboration, increased productivity, and the creation of a positive work environment. Studies show that organisations in which employees demonstrate high levels of OCB tend to be more resilient to stress and conflict, and achieve higher levels of employee satisfaction [83,84,85]. Specifically, supporting employee well-being is key to developing a positive work environment and increasing organisational citizenship behaviour (OCB), thereby contributing to the organisation’s overall effectiveness [86,87,88,89].

## 5. Conclusions

This study directly addressed a research gap by moving beyond the universalist approach and providing the first systematic empirical evidence on how organizational type and work position statistically differentiate the perception of engagement ante-cedents in the Slovak healthcare sector. Our findings, therefore, lay the foundation for the shift towards differentiated management, offering prescriptive insights necessary to develop targeted HRM strategies that enhance engagement and long-term sustainability of healthcare workforce.

The conclusions of this study provide empirical insights regarding the antecedents of employee engagement and well-being within the Slovak healthcare sector, thereby filling an existing geographical and empirical gap in Central European literature.

The theoretical contribution of this study primarily lies in its identification and empirical structuring of key engagement antecedents, which were grouped into three distinct factors. Through this process, the study provides a validated factor structure specific to the Slovak context. It was also demonstrated that employees’ perception of these factors is statistically influenced by the type of organization and the employees’ work position. These significant findings advance the existing knowledge regarding sociodemographic variability in the healthcare sector.

The practical contribution of this study lies in offering concrete recommendations for HR departments by application of differentiated management approaches, as it specifies what is important (the factors) and for whom it is most important (organization type and work position) within Slovak healthcare. More specifically, the practical benefit consists of identifying the need for differentiated management based on the type of organization and identifying a differentiated need for employee involvement based on their work position.

Although this research study provides valuable insights, like all studies, it also has its limitations. The main limitation of this research is considered to be its limited geographical scope. Furthermore, even though the research sample is relatively heterogeneous, it may not fully capture all the nuances of the entire healthcare sector. A larger and more diverse sample with the possibility of collecting longitudinal data could provide a more comprehensive understanding of the complex interplay between well-being and employee retention in the healthcare sector. Future research should be focused on the examination of specific factors contributing to the negative perception of organizational commitment, job meaningfulness, and job satisfaction within different types of healthcare organizations and work positions. Qualitative research methodologies, such as interviews and focus groups, could provide valuable insights into the lived experiences of employees and contribute to a deeper understanding of these complex issues. By implementing these recommendations, the growth of employee engagement and well-being can be supported by healthcare organizations in Slovakia, which will lead to an improvement in organizational effectiveness and, ultimately, to enhanced patient care.

## Figures and Tables

**Table 1:** Eigenvalues of correlation matrix.

Value Number	Eigenvalues of Correlation Matrix, and Related Statistics (Active Variables Only)			
	Eigenvalue	% Total Variance	Cumulative Eigenvalue	Cumulative %
1	9.853468	54.74149	9.85347	54.7415
2	1.271232	7.06240	11.12470	61.8039
3	1.019160	5.66200	12.14386	67.4659
4	0.837035	4.65020	12.98090	72.1161
5	0.726384	4.03547	13.70728	76.1516
6	0.597913	3.32174	14.30519	79.4733
7	0.483610	2.68672	14.78880	82.1600
8	0.451173	2.50652	15.23998	84.6665
9	0.405256	2.25142	15.64523	86.9180
10	0.375205	2.08447	16.02044	89.0024
11	0.336487	1.86937	16.35692	90.8718
12	0.313145	1.73969	16.67007	92.6115
13	0.296283	1.64602	16.96635	94.2575
14	0.243122	1.35068	17.20947	95.6082
15	0.226035	1.25575	17.43551	96.8639
16	0.210046	1.16692	17.64555	98.0309
17	0.189132	1.05073	17.83469	99.0816
18	0.165314	0.91841	18.00000	100.0000

Source: own elaboration.

**Table 2:** Factor loadings extraction from PCA.

Variable	Factor Loadings (Varimax Normalized) Extraction: Principal Components (Marked Loadings Are > 0.700000)		
	Factor 1	Factor 2	Factor 3
PA_1	0.571540	0.464758	0.319909
PA_2	0.511910	0.592615	0.271631
PA_3	0.645115	0.431292	0.052068
PA_4	0.333800	0.751369	0.133296
PA_5	0.352668	0.756873	0.048166
PA_6	0.394348	0.747626	0.135004
PA_7	0.112367	0.768509	0.303793
PA_8	0.737008	0.411117	0.069006
PA_9	0.802443	0.244463	0.145500
PA_10	0.766068	0.263942	0.215896
PA_11	0.354525	0.631954	0.329894
PA_12	0.741196	0.283659	0.316683
PA_13	0.660605	0.313888	0.443738
PA_14	0.579889	0.123383	0.553935
PA_15	0.403600	0.077617	0.709121
PA_16	0.500003	0.373187	0.191714
PA_17	0.670308	0.346721	0.298616
PA_18	0.046534	0.343964	0.780606
Expl.Var	5.494874	4.316013	2.332973
Prp.Totl	0.305271	0.239778	0.129610

Source: own elaboration.

**Table 3:** ANOVA Univariate Tests of Significance for Factor 1.

Effect	Univariate Tests of Significance for Factor 1. Sigma-Restricted Parameterization Effective Hypothesis Decomposition; Std. Error of Estimate: 0.8923				
	SS	Degr. of Freedom	MS	F	*p*
Intercept	0.0109	1	0.010904	0.013696	0.906990
Gender	2.0235	1	2.023514	2.541655	0.112929
Age	2.6787	5	0.535745	0.672929	0.644569
Education	5.2363	3	1.745448	2.192387	0.091199
Organizational size	0.4449	2	0.222436	0.279393	0.756625
Type of the organization	9.0504	2	4.525191	5.683911	0.004153
Work position	10.0030	7	1.428994	1.794903	0.091964
Error	122.6056	154	0.796140		

Source: own elaboration.

**Table 4:** ANOVA Univariate Tests of Significance for Factor 2.

Effect	Univariate Tests of Significance for Factor 2. Sigma-Restricted Parameterization Effective Hypothesis Decomposition; Std. Error of Estimate: 0.9280				
	SS	Degr. of Freedom	MS	F	*p*
Intercept	1.3751	1	1.375097	1.596731	0.208277
Gender	0.3960	1	0.396042	0.459874	0.498700
Age	1.7611	5	0.352216	0.408985	0.842022
Education	1.2669	3	0.422298	0.490363	0.689480
Organizational size	1.4851	2	0.742551	0.862234	0.424246
Type of the organization	0.3459	2	0.172955	0.200832	0.818264
Work position	30.3089	7	4.329838	5.027708	0.000037
Error	132.6241	154	0.861195		

Source: own elaboration.

**Table 5:** ANOVA Univariate Tests of Significance for Factor 3.

Effect	Univariate Tests of Significance for Factor 3. Sigma-Restricted Parameterization Effective Hypothesis Decomposition; Std. Error of Estimate: 0.9639				
	SS	Degr. of Freedom	MS	F	*p*
Intercept	0.7481	1	0.748080	0.805097	0.370983
Gender	0.0773	1	0.077284	0.083175	0.773431
Age	9.7708	5	1.954165	2.103107	0.067970
Education	0.9701	3	0.323354	0.348000	0.790645
Organizational size	1.7743	2	0.887145	0.954761	0.387185
Type of organization	2.4792	2	1.239622	1.334103	0.266441
Work position	8.5259	7	1.217982	1.310814	0.248712
Error	142.1645	153	0.929180		

Source: own elaboration.

## Data Availability

Dataset available on request from the authors. The raw data supporting the conclusions of this article will be made available by the authors on request. The data supporting the reported results, which were analyzed and generated during the study, are available upon reasonable request from the authors of this publication via their corresponding email addresses.
